# Implementation of an Acute Tonsillitis Protocol in a District General Hospital: A Two-Cycle Audit

**DOI:** 10.7759/cureus.98141

**Published:** 2025-11-30

**Authors:** Noon Nassr, Maria Sikander, Victoria Ward

**Affiliations:** 1 Otolaryngology, Pinderfields Hospital, Wakefield, GBR

**Keywords:** acute tonsillitis, clinical audit, ent emergency, patient-flow, protocol implementation

## Abstract

Background

Acute tonsillitis is a common presentation in ear, nose, and throat (ENT), placing a considerable burden on emergency and inpatient services due to the high volume of admissions and resource use. At our district general hospital, the absence of a formalised pathway contributed to inconsistent reassessment practices and prolonged admissions. This audit evaluated whether implementing a standardised acute tonsillitis protocol could improve the timeliness of clinical review and support more efficient inpatient management.

Methods

A two-cycle audit was conducted over identical three-month periods (September to November 2023 and 2024). Adult patients (≥18 years) referred to ENT with a clinical diagnosis of tonsillitis were included. A structured protocol was introduced, emphasising reassessment within six hours to identify suitability for discharge on oral therapy. Implementation included departmental teaching, protocol dissemination, and the use of visual prompts. Data were collected retrospectively in Cycle 1 and prospectively in Cycle 2. Outcomes included time to second review, length of stay (LOS), and two-week readmission rates. Analyses were performed using Mann-Whitney U, Fisher’s exact test, and linear regression.

Results

A total of 49 patients were included in Cycle 1 and 54 in Cycle 2. The median time to second review decreased by 3.25 hours (9.0 to 5.75 hours, p = 0.031) following protocol implementation. The median LOS reduced by six hours (24.0 to 18.0 hours), though not statistically significant (p = 0.083). Among patients with a recorded second review, no significant association was found between review timing and LOS (Cycle 1: β = 0.89, p = 0.24, Cycle 2: β = −0.38, p = 0.55). The two-week readmission rate increased from 6.1% to 11.1%, though this was not statistically significant (p = 0.49).

Conclusion

Implementation of a standardised tonsillitis protocol improved the consistency and timeliness of inpatient reassessment and showed a clinically meaningful trend toward reduced LOS. While not statistically significant, the observed operational gains may support more efficient bed use in resource-constrained services. Based on the observed reduction in length of stay and typical monthly admission volumes, this corresponds to an estimated four to five bed-days released per month. Ongoing monitoring, staff education, and re-audit are required to ensure safe, sustained adoption and to further evaluate the pathway’s longer-term clinical and service impact.

## Introduction

Acute tonsillitis is a common ear, nose, and throat (ENT) presentation in emergency departments, accounting for 70,658 admissions in 2023-2024 [1]. With the average daily cost of a non-elective National Health Service (NHS) hospital bed estimated at £901 [2], this represents a significant financial and operational burden on both ENT and emergency services. For district general hospitals with limited bed capacity and high emergency demand, even short, potentially avoidable admissions can contribute to crowding, delays in elective activity, and increased staff workload.

At our district general hospital, there was no formalised local pathway for acute tonsillitis prior to this project. Admission and discharge decisions were largely clinician-dependent, reassessment times were inconsistent, and documentation of second reviews was variable. As a result, patients were frequently retained until the following day’s ward round before a discharge decision was made, even when clinically stable. This contributed to unnecessarily prolonged admissions and inefficient use of inpatient beds. These challenges were particularly evident during periods of high emergency demand, when ENT on-call teams balance ward work, emergency referrals, and theatre commitments.

The Portsmouth Tonsillitis Protocol, developed by Bird et al., was designed to standardise the management of patients presenting to emergency departments with acute tonsillitis. Its implementation led to a 54% reduction in hospital admission rates and a 22-hour reduction in average inpatient stay per patient [3]. Because time to review and length of stay (LOS) were also key drivers of inefficiency in our services, these were selected as the primary process measures for evaluation. Earlier reassessment provides an opportunity to identify patients suitable for discharge on oral therapy, potentially shortening admission duration and improving bed availability.

The primary objective of this audit was to evaluate the impact of implementing a standardised acute tonsillitis protocol within our district general hospital, with the aim of reducing inpatient length of stay through timely reassessment and earlier discharge. Secondary objectives included assessing two-week readmission rates to ensure that any operational gains did not compromise patient safety. The intervention was evaluated using a two-cycle audit comparing outcomes before and after implementation.

## Materials and methods

A retrospective audit was conducted over a three-month period (1st September to 30th November 2023) and included patients aged ≥18 years referred to the ear, nose, and throat (ENT) with a primary diagnosis of acute tonsillitis confirmed by the assessing ENT clinician. Acute tonsillitis was defined using clinical criteria consistent with the National Institute for Health and Care Excellence (NICE), including tonsillar erythema or exudate, fever, odynophagia, and tender cervical lymphadenopathy [4]. Exclusion criteria included suspected or confirmed peritonsillar abscess, an alternative diagnosis following senior review, incomplete documentation preventing assessment of key outcomes, and patients discharged directly from the emergency department without ENT review.

Patients were identified through daily ENT on-call referral logs and electronic patient records. For the retrospective cycle, data were extracted using a predefined proforma to ensure consistency with the prospective cycle. Variables collected included demographics, timing of reviews, length of stay (LOS), treatment received, and readmission within two weeks. Time to second review was obtained from the documented review time; if this was not recorded, the timing was determined from the timestamp of the clinical entry describing the second review.

A prospective second audit cycle was conducted over the same three-month period in 2024 (1st September to 30th November 2024) using identical inclusion and exclusion criteria. Data were collected in real time using the same predefined proforma to ensure consistency with the retrospective cycle. Formal compliance monitoring was not feasible during routine clinical practice. Therefore, documentation of review timing and discharge decisions was used as a pragmatic indicator of adherence to the protocol. Staff engagement was supported through teaching and dissemination, but quantitative measures of uptake were not collected.

**Figure 1:**
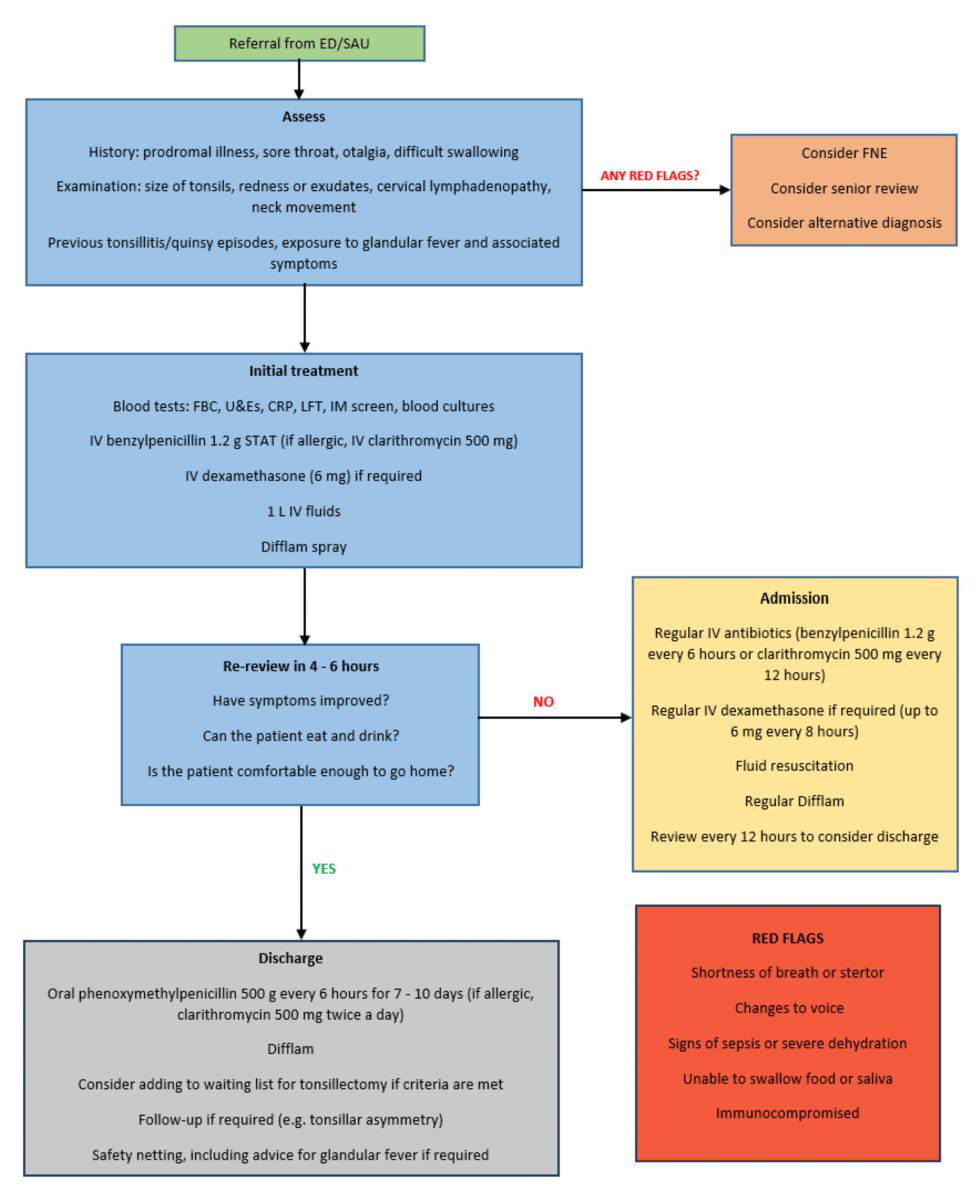
Acute tonsillitis management protocol for adults (≥18 years) ED: Emergency Department; SAU: Surgical Assessment Unit; FNE: Flexible Nasendoscopy; FBC: Full Blood Count; U&Es: Urea and Electrolytes; CRP: C-reactive Protein; LFT: Liver Function Tests; IM: Infectious Mononucleosis; IV: Intravenous; g: gram; mg: milligram; L: Litre.

A standardised acute tonsillitis protocol (Figure 1) was introduced with the aim of ensuring clinical reassessment within six hours of initial review. The protocol outlined criteria for intravenous therapy, indications for senior review, and discharge criteria. Implementation included teaching sessions delivered to all rotating ENT junior doctors at the start of each four-month rotation, inclusion of the protocol in the ENT departmental handbook, dissemination to emergency department (ED) clinicians, and posters displayed in the ENT office.

Patients were managed according to the National Institute for Health and Care Excellence (NICE) [5] and ENT UK Adult Acute Severe Sore Throat Management guidelines [6]. Initial management included intravenous antibiotics in line with local antimicrobial protocols and a single dose of intravenous dexamethasone, supported by evidence demonstrating its efficacy in symptom relief [7]. Patients presenting with red flag symptoms (Figure 1) were considered for senior review, alternative diagnosis, and further investigations such as fibreoptic nasoendoscopy (FNE) [8]. 

This audit was registered with the hospital Clinical Audit Department and conducted as a service-evaluation project. Ethical approval was not required. All data were pseudonymised prior to analysis. As this was a service-evaluation audit, no formal sample size calculation or power analysis was undertaken. Both audit cycles included all eligible patients presenting during the defined periods, providing a complete consecutive sample reflective of routine clinical activity. Data were collated in Microsoft Excel 365 and analysed in R (version 4.5.1). Non-parametric continuous variables (review time and LOS) were analysed using the Mann-Whitney U test. Categorical variables were compared with Fisher’s exact test. Simple linear regression assessed associations between time to second review and LOS. Statistical significance was defined as p < 0.05.

## Results

**Figure 2:**
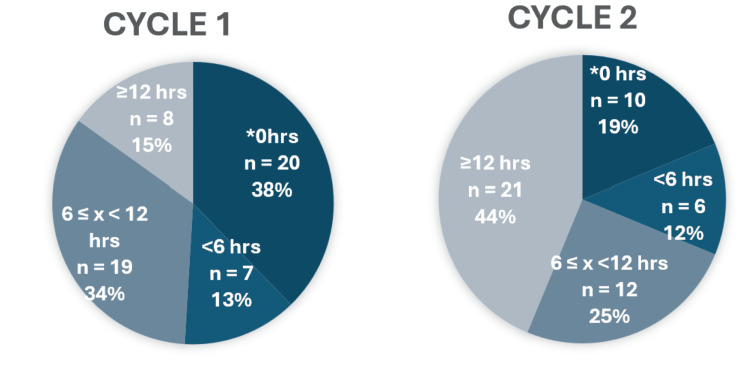
Distribution of time to second review among patients admitted with acute tonsillitis before (Cycle 1) and after (Cycle 2) protocol implementation hrs: hours. * 0hrs = no second review documented

A total of 49 patients were reviewed in Cycle 1 (21 males, 28 females, mean age 27.2 years) and 54 in Cycle 2 (24 males, 30 females, mean age 26.7 years). The distribution of time to the second review is shown in Figure 2. In Cycle 1, 19% (n = 10) of patients were not reviewed a second time, 12% (n = 6) were reviewed within six hours, 25% (n = 12) between six and 12 hours, and 44% (n = 21) waited ≥12 hours for reassessment. Following protocol implementation (Cycle 2), 38% (n = 20) of patients were not reviewed a second time, 13% (n = 7) were reviewed within six hours, 34% (n = 19) between six and 12 hours, and only 15% (n = 8) waited ≥12 hours. Patients who were discharged without a documented second review typically met discharge criteria at initial assessment and were deemed suitable for oral antibiotics and outpatient management.

The median time to second review significantly decreased from 9.0 hours in Cycle 1 to 5.75 hours in Cycle 2 (p = 0.031), representing an absolute reduction of 3.25 hours. Patients without a documented second review were excluded from inferential analyses. The median length of stay (LOS) decreased from 24.0 hours in Cycle 1 to 18.0 hours in Cycle 2; an absolute reduction of 6 hours; however, this difference did not reach statistical significance (p = 0.083). While modest, this change represents a clinically relevant shift for a short-stay ENT condition, with potential implications for bed availability during periods of operational pressure.

Among patients with a recorded second review, no significant association was observed between review timing and LOS in either audit cycle. In Cycle 1, a weak positive trend was noted (n = 39, β = 0.89, 95% CI -0.61 - 2.39, p = 0.24), suggesting that longer waits for reassessment tended to accompany longer admissions, though not significantly. In Cycle 2, the relationship was negligible (n = 34, β = -0.38, 95% CI -1.67 - 0.90, p = 0.55), indicating that after protocol implementation, LOS was not meaningfully influenced by the timing of the second review. Subgroup analysis was limited by sample size and therefore not undertaken.

The two-week readmission rate was increased from 6.1% (3/49) in Cycle 1 to 11.1% (6/54) in Cycle 2, however, this difference was not statistically significant (p = 0.49). Among the readmissions, documented reasons included persistent odynophagia, inadequate oral intake, and concerns regarding symptom progression. No cases required operative intervention, and no consistent pattern of severity or clinical features was observed. Owing to the small absolute numbers, subgroup analysis did not demonstrate any clear predictors of re-attendance.

## Discussion

This audit evaluated the impact of a standardised acute tonsillitis protocol on inpatient management within a district general hospital. Implementation of the protocol was associated with a significant reduction in time to second review and a trend towards shorter hospital stay. These findings support the role of structured clinical pathways in improving the consistency of care for common ENT presentations. Prior to implementation, reassessment frequently occurred after prolonged delays, with 41% of patients waiting ≥12 hours for a second review. Following the introduction of the protocol, only 15% of patients waited ≥12 hours, and the median time to reassessment decreased by 3.25 hours. This improvement likely reflects clearer expectations for review timing and better integration of reassessment into the routine workflow. Practical factors may also have contributed, including increased awareness among junior doctors, improved handover of review responsibilities, and the use of visual prompts such as posters in the ENT office. These process changes resemble the improvements described by Bird et al., whose Portsmouth protocol demonstrated marked reductions in admissions and inpatient stay [3]. Comparable studies have also shown that early senior review and standardised discharge criteria can significantly improve flow in ENT and other acute specialities [9,10].

Although the reduction in median LOS did not reach statistical significance, the observed six-hour reduction is operationally meaningful for a short-stay condition. In district general hospitals with constrained bed capacity, small reductions in LOS can accumulate to create measurable improvements in bed turnover and emergency flow, leading to meaningful operational and financial benefits [9]. The lack of statistical significance may reflect limited sample size, but it may also indicate that LOS is influenced by factors beyond the timing of clinical review. These may include staffing patterns (e.g., reduced senior presence overnight), bed availability, patient complexity, delays in antibiotic preparation, and competing demands on the on-call ENT team. Previous research has highlighted that LOS is often determined by a combination of patient, organisational, and staffing factors, including access to senior decision makers, rather than a single process measure [11-13]. 

Based on the median six-hour reduction in length of stay and the monthly admission volume across both audit cycles, this equates to approximately four to five bed-days released per month. While modest, this represents a meaningful operational gain for a short-stay ENT condition within a district general hospital, particularly during periods of high bed pressure. In addition to these operational benefits, more timely reassessment may also support earlier identification of clinical deterioration, reduce unnecessary overnight admissions, and improve patient experience by facilitating clearer treatment plans and faster discharge when appropriate.

The increase in two-week readmission rates from 6.1% to 11.1% did not reach statistical significance and may reflect normal variation. No consistent pattern in readmission indication was observed. Previous evaluations have shown that protocol-driven management can reduce admissions and readmissions safely [14,15], but the present audit was not powered to detect differences in safety outcomes. Ongoing monitoring is therefore essential to ensure that earlier discharge does not inadvertently contribute to increased reattendance.

Strengths of this audit include its direct applicability to everyday ENT practice, the use of a two-cycle design allowing temporal comparison, and a clearly defined intervention. However, several limitations must be acknowledged. These include the single-centre design, relatively small sample size, and variation in documentation among rotating junior staff. The audit’s descriptive nature prevents adjustment for confounders, and the absence of formal compliance monitoring limits the ability to attribute changes solely to the protocol. In addition, variation in initial clinical severity may have contributed to differences in outcomes, although the audit design did not allow for formal severity stratification. Despite these limitations, the audit provides practical insight into the feasibility of implementing a time-based reassessment pathway and highlights areas for further service improvement.

Sustaining improvement will require continued education, reinforcement of review expectations, and periodic re-audit in line with Healthcare Quality Improvement Partnership (HQIP) recommendations [16]. Larger, multi-centre studies may help clarify the relationship between early reassessment, LOS, and readmission, and allow more robust evaluation of cost efficiency and clinical outcomes.

## Conclusions

This audit demonstrates that a standardised tonsillitis protocol can improve the consistency and timeliness of patient reassessment within a district general hospital. While the reduction in length of stay did not reach statistical significance, the observed trend suggests that structured review processes may help support more efficient inpatient management. Ongoing monitoring, continued education, and periodic re-audit will be important to ensure sustained adherence and to evaluate the wider clinical and operational impact of this pathway. Given the simplicity and low-resource nature of this intervention, similar pathways could be feasibly adopted or evaluated across other centres, supporting wider efforts to standardise acute tonsillitis management within ENT services.
